# Rotations of teeth—a systematic review

**DOI:** 10.3389/froh.2024.1484020

**Published:** 2024-12-06

**Authors:** Ramya Parthiban, Vignesh Kailasam, Nivetha Shree Venkatasamy

**Affiliations:** Sri Ramachandra Medical College and Research Institute, Sri Ramachandra Institute of Higher Education and Research, Chennai, India

**Keywords:** classification, anterior rotation, premolar rotation, molar rotation, retention, stability, relapse

## Abstract

**Background:**

Rotations are frequently evaluated through various assessment methods of crowding and arch dimension, with relatively few studies discussing the extent or direction of rotations and even fewer addressing the reliability of such assessments. This systematic review aims to comprehensively analyze existing classification systems for rotated teeth and assess rotation in anterior and posterior teeth, its clinical applicability, and its impact on retention and relapse.

**Search methods:**

Two investigators conducted a comprehensive search in six databases, namely, PubMed, Scopus, Ovid, LILACS, Web of Science, and Cochrane CENTRAL, up to 28 March 2024. No specific start date was defined to ensure the inclusion of all relevant studies from the inception of each database, maximizing the comprehensiveness of our review. The search criteria included retrospective studies and the inclusion criteria were patients who were assessed for rotation in any age group. The exclusion criteria were patients who had undergone orthodontic treatment, who had fractured restorations or crowns, or who had any other tooth anomaly.

**Data collection and analysis:**

In total, 10 studies satisfying the inclusion criteria were included and 9 provided quantitative outcomes for the rotation of various teeth, while the remaining study offered qualitative results. The risk of bias assessment was performed with the help of the Newcastle–Ottawa quality assessment tool.

**Results:**

The skeletal Class II and Class III groups exhibited similar average positions of the first molar. Upper molar rotation was primarily observed in dental Class II patients, with a higher mesial rotation angle of 78.6°. Only one study measured the rotation for all permanent teeth. Seven studies used the mid-palatal raphe as the reference line for measuring molar rotation. No gender differences were found. It was found that there was no statistical significance in the mean values of molar rotation for the right and left sides as well as the maxillary and mandibular arches. The incisors demonstrated the highest degree of rotation (7.4°–20.2°), while the premolars and canines exhibited a slightly lower degree of rotation (3.3°–9.2°). In contrast, the molars displayed the lowest degree of rotation (0.8°–7.4°).

**Conclusion:**

After reviewing all the studies, it was found that there is no adequate classification system to assess the rotation of anterior teeth and mandibular teeth. A universally accepted classification of tooth rotation, including a common reference line, is needed. The existing systems for posterior teeth need to be standardized and have a clinical utility to be widely accepted.

**Systematic Review Registration:**

https://www.crd.york.ac.uk/prospero/display_record.php?ID=CRD42024524654, PROSPERO (CRD42024524654).

## Introduction

The rotation of teeth is a common dental anomaly characterized by the misalignment of a tooth along its long axis, resulting in a deviation from its normal position within the anterior and posterior regions of the dental arch (1). Studies conducted in various populations have reported prevalence rates ranging from 5% to 30%, depending on the criteria used for defining rotated teeth and the characteristics of the study population (2). Rotated teeth can manifest in various degrees and directions, complicating their assessment and treatment (3).

The exact etiology of tooth rotation is unknown, though it is more likely to have a developmental origin. Displacement of the dental follicle from its path during eruption can cause tooth rotations (3). Various genetic, developmental, environmental, occlusal, and orthodontic factors contribute to this condition. Genetic predisposition, such as hereditary dental crowding or jaw size discrepancies, may lead to rotations as the teeth adjust to limited space within the arch (4). Developmental factors, such as malposition of the tooth bud or disruptions in the eruption sequence, can also influence tooth positioning. Environmental causes, such as the premature loss of primary teeth, trauma to the jaw, or prolonged oral habits (e.g., thumb sucking or tongue thrusting), generate imbalanced forces that result in rotations (5). In some cases, orthodontic treatment itself can lead to rotations if there is improper planning or a lack of retainers, causing relapse. Periodontal problems, such as bone loss or localized infections, weaken the supporting structures, enabling teeth to migrate and rotate (6). In addition, impacted third molars can exert pressure on adjacent teeth, and congenitally missing teeth may leave space that allows others to rotate into abnormal positions. A tooth's location within the arch can also be affected by unusual eruption sequences, inadequate space, and excessive force from the tongue and lips, displacing it from its ideal position (6). Various methods have been developed to assess tooth rotation by relating teeth to their correct positions in the dental arch.

A rotated tooth can create numerous esthetic and psychological problems for a patient. Anterior tooth rotation is more noticeable and impacts smile esthetics. Posterior tooth rotation affects occlusal stability and chewing function, leading to issues such as uneven wear or discomfort during mastication (1–5). The correction of orthodontically rotated teeth is challenging because it tends to develop some degree of post-retention relapse (6). Collating the various rotation assessment systems will aid orthodontists in accurately diagnosing and planning appropriate interventions. This would facilitate communication among professionals and enhance research comparability. This systematic review aims to comprehensively analyze existing classification systems for rotated teeth; by synthesizing the available literature, we seek to provide insights into the strengths, limitations, and clinical applicability of these classification frameworks.

## Aims and objective

The aim of this study was to collate and assess the various classification systems for tooth rotation and compare them in terms of clinical applicability and diagnostic consistency in the general population.

### PICO

**Population (P):** Patients with rotated teeth
**Intervention (I):** Classification systems for dental rotations
**Comparison (C):** Comparison between different classification systems
**Outcome (O):** Clinical implications of assessment of rotation for retention and relapse

## Materials and method

### Protocol and registration

**Figure 1:**
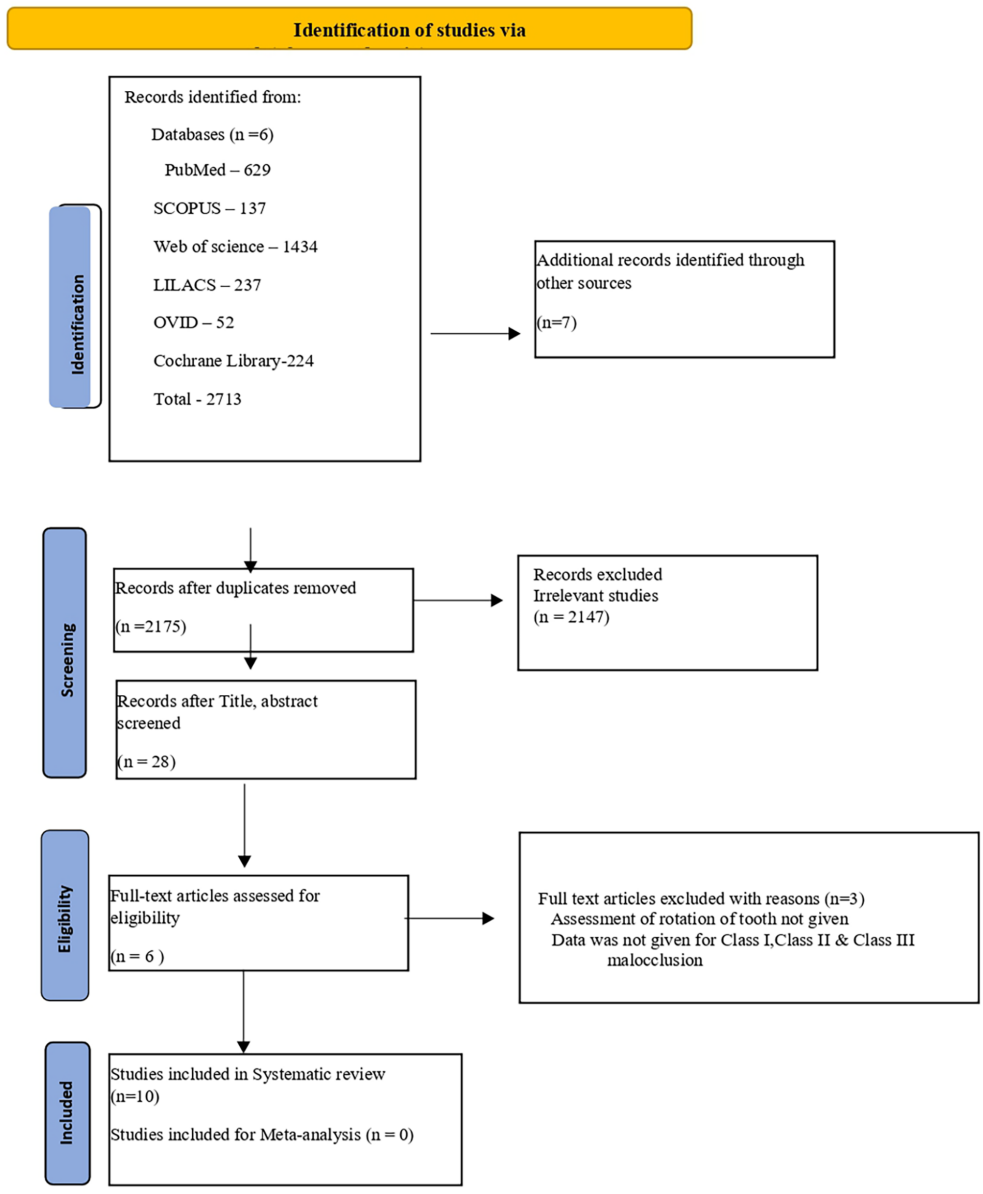
PRISMA flow chart.

This systematic review was conducted in accordance with the Preferred Reporting Items for Systematic Reviews (PRISMA) guidelines (Figure 1) (7) and has been registered with PROSPERO under the ID number CRD42024524654.

### Eligibility criteria

#### Inclusion criteria

• Patients with skeletal Class I, II, and III malocclusions across all age groups.
• Good periodontal health.
• Patients with cleft lip and palate.
• No underlying medical history.

#### Exclusion criteria

• Patients with a prior history of orthodontic treatment.
• Presence of fractured restorations or crowns.
• Any tooth anomaly.
• Premature or delayed exfoliation/extraction of primary teeth that may cause tooth rotation.

### Information sources and search strategy

A systematic search was performed across six electronic databases: Medline (via PubMed), Cochrane Library, Scopus, Web of Science, OVID, and LILACS. The search approach used MeSH (Medical Subject Headings) and the Boolean Operators “AND” and “OR” up to 28 March 2024. No specific start date was defined to ensure the inclusion of all relevant studies from the inception of each database, maximizing the comprehensiveness of our review. However, the search was limited to articles written in English. A manual search of the reference lists of the included articles was also done (Table 1). A gray literature search was also done in accordance with the Cochrane Handbook's guidelines for systematic reviews (17). Specifically, a hand search of reference lists from selected individual studies and a hand search in four international orthodontic journals (AJODO, EJO, The Angle Orthodontist, Progress in Orthodontics) were conducted, enabling the inclusion of additional gray literature. The details of this search are documented in the PRISMA flowchart.

**Table 1:** Study characteristics and results.

Author and year	Sample size	Participants	Methods of assessment	Outcome
Henry (1956) (8)	115 models	1. 20 normal occlusion participants 2. 25 Class I malocclusion participants (4 premolar extraction) 3. 30 Class II division 1 malocclusion participants 4. 20 Class II division 1 subdivision participants 5. 20 Class II division 2 malocclusion participants	Angles formed between the midline passing through palate and mesiobuccal and mesiopalatal cusp tip of molar (M and M’ angle)	1. Normal: 10.3° (R), 12° (L) 2. Class I extraction: 19.5° (R), 15.5° (L) 3. Class II division 1: 20° (R), 17.6° (L) 4. Class II division1 subdivision: 18.8° (R), 16° (L) 5. Class II division 2: 19.5° (R), 16.9° (L)
Lamons and Holmes (1961) (9)	90 models	Group 1: normal occlusion; Group 2: Post-normal occlusion; Group 3: prematurely extracted deciduous molar	Line joining mesiobuccal and mesiolingual cusps and a midline through the median raphe	Group 1: 59.78° (R) and 56.71° (L) Group 2: 52.12° (R) and 51.30° (L) Group 3: 45 0.55° (R) and 47 0.03° (L)
Ricketts (1969) (10)	Sample size was not mentioned	Participants were not described	The mesiopalatal and distobuccal cusps, the first molar, and the contralateral canine are measured	This is a good method for checking the proper distal rotations of the upper first molar in evaluating mesial drift in a malocclusion
McMullan and Richardson (1991) (11)	20 cases	Near normal occlusion	Line joining canines and posterior teeth and line joining the buccal and palatal cusps of the premolar (90-n degrees) • Positive sign for mesiobuccal rotation • Negative sign for mesiolingual rotation • Upper and lower all premolars	14 − +4.1°, 15 − −1.4°, 24 − +7.6°, 25 − +2.7°, 34 − −12.9°, 35 − −5.5°, 44 − −9.8° Upper first premolars, +4.1° (R) and +7.6° (L), had the highest rotation and the least was the first lower premolar, −12.9° (R) and −9.8° (L)
Dahlquist et al. (1996) (12)	84 cases	Group 1: normal occlusion (ideal occlusion); Group 2: treatment group	Cusp tips of molars and reference lines used by Orton et al., Friel et al., Henry and Ricketts.	Angle 1: 4.3° Angle 2: 2.5° Angle 3: 2.0°
Hansen et al. (1997) (13)	94 models	Normal untreated patients	Rotation of maxillary and mandibular first molars and maxillary first premolars Midline through the palate and a line tangent and the buccal cusp tip of the molars	Maxillary first molar: 14.08° (R), 12.8° (L) Maxillary first premolar: 6.9° (R), 6.6° (L) Mandibular first molar: 19.8° (R), 21.9° (L)
Junqueira et al. (2011) (14)	Group 1: 60 models Group 2: 120 models (scanned and digitized)	Group 1: normal occlusion; Group 2: Class II division 1 malocclusion	1. Angle 1: A line through the midpalatine suture (Line A), a line through the mesiobuccal cusp and distobuccal cusp (B) 2. Angle 2: A line through the midpalatine suture (A), a line through the mesiobuccal cusp and mesiolingual cusp (C) 3. Angle 3: A line through the mesiobuccal cusp and mesiolingual cusp (B) and a line tangent through the premolars (D)	Group 1 Angle 1: 10.07° Angle 2: 61.54° Angle 3: 12.69° Group 2 Angle 1: 14.97° Angle 2: 57.44° Angle 3: 5.93° Class II division 1 malocclusion patients present with greater mesiopalatal rotation of the maxillary molars
Vermeulen et al. (2012) (5)	10 models of both maxilla and mandibles (cast photograph)	Class I and Class II malocclusions, all treated with non-extraction T1: pre-treatment; T2: post-treatment; T3: after removal of retention appliance	Arch form and line through or parallel to the most occlusal points of the buccal cusp. Arch form drawn using seven points on the digital photograph The locations of these points were as follows: (1) Distal from the first right molar, in a way that the extending line will go through or be parallel to the most occlusal points of the buccal cusps; (2) Between the two right premolars, in a way that the extending line will go through or be parallel to the most occlusal points of the buccal cusps of the premolars and the canine; (3) Mesial from the right canine, in a way that the extending line will go through the most incisal point of the canine and the incisal edges of the incisors; (4) Between the two central incisors, in a way that the extending line will go through the incisal edges of the incisors; and (5–7) the same points as above but in the contralateral quadrant. Rotation was assessed from central incisor to first molar by placing points on the mesial and distal ideal contact points of the tooth in a way that the line through these points was perpendicular to the buccolingual axis of the tooth. The angle of rotation was the angle between the mesiodistal line and the line, indicating the ideal position on the arch.	Reliability of rotation assessment was conducted
Scanavini et al. (2014) (15)	60 models 27 females and 33 males, aged between 12 and 21 years	Class II division 1 malocclusion	The sagittal position of the molars was determined by positioning the casts onto the device, considering the mid-palatal suture as a symmetry reference, and then measuring the distance between the mesial marginal ridge of the most distal molar and the mesial marginal ridge of its counterpart With regard to the degree of rotation of the upper molar, the distance between landmarks on the mesial marginal ridge was measured	Molar rotation, measured in millimeters, represented 1⁄4 of Class II
de Oliveira Viganó et al. (2016) (16)	100 models	Non-treated groups 1, 2, 3, and 4; Group 1: Class I (20); Group 2: Class II (Dental); Group 3: Class II (Skeletal); Group 4: Class III (Skeletal); Group 5: Class I (treated)	Angle: A line passing through the mid-palatal raphe and a line passing through the distobuccal cusp and mesiolingual cusp	Group 1: Mean 71.23° Group 2: Mean 78.95° Group 3: Mean 74.45° Group 4: Mean 73.83° Group 5: Mean 67.46° Skeletal classes II and III showed similar mean positions for the maxillary first molar (50% of the patients) Mesiopalatal rotation of the U1st M (78.6°) had the highest mean in Class II (dental)

### Study selection

The study selection was conducted by two investigators (RP and NV) in two phases. Initially, they independently screened articles based on the research question and eligibility criteria. Titles and abstracts were reviewed first, followed by a full-text review if the abstract and title provided incomplete information. In addition, hand-searching was performed to ensure no relevant articles were missed. When information was unclear or missing, the authors were contacted. The final pool of articles was assessed for eligibility for qualitative and quantitative reviews. Discrepancies between the two reviewers were resolved through discussion, and any remaining disagreements were settled by a third reviewer (VK).

### Data collection process and data items

The two reviewers (RP and NV) independently extracted relevant data. If any questions arose regarding a specific study, the lead author (VK) was contacted for clarification. Each reviewer initially entered the data into a Microsoft Word document separately and then discussed discrepancies to reach a consensus. After retrieving 2,713 studies from the six databases, Zotero (version 6.0.37, Corporation for Digital Scholarship, USA) was used to remove 538 duplicate records, reducing the number of studies for further screening to 2,175. Following title and abstract screening, 22 studies were excluded, and 6 studies were initially selected for full-text evaluation. However, only three articles met the inclusion criteria after full-text evaluation. In addition to these three studies, seven more articles were identified through a hand search in four orthodontic journals and the reference lists of individual studies. These seven articles also satisfied the inclusion criteria and were included in the final selection. As a result, a total of 10 studies were incorporated into this systematic review. Each included study was individually examined, extracting data regarding the author's name, study design, sample size, participants, and assessment methods (Table 1). The finalized data were shared with the senior reviewer for approval.

A risk of bias (RoB) assessment was performed using the Newcastle–Ottawa Scale (NOS), covering the domains of quality of reporting, external validity, internal validity, and power (Table 2). The studies were graded as poor, moderate, or good quality based on this scale. Eight studies were graded as moderate and one as good. Disagreements during the risk of bias assessment were resolved through discussion with a third reviewer. The quality assessment scores for the Newcastle–Ottawa Scale are 6–9 for good quality, 3–5 for moderate quality, and 0–2 for poor quality.

**Table 2:** Risk of bias assessment.

Quality evaluation	de Oliveira Viganó et al., 2016 (16)	Scanavini et al., 2014 (15)	Vermeulen et al., 2012 (5)	Junqueira et al., 2011 (14)	Hansen et al., 1997 (13)	Dahlquist et al., 1996 (12)	McMullan and Richardson, 1991 (11)	Ricketts et al., 1969 (10)	Lamons and Holmes, 1961 (9)	Henry, 1956 (8)
Selection										
Is the case definition adequate?	*	*	*	*	*	*		*	*	*
Representativeness of the rotation assessment group	*	*	*	*	*	*		*	*	*
Selection of a control group										
Definition of controls						*				
Comparability										
Comparability of participants in the treatment groups and control groups										
Exposure										
Ascertainment of a rotation assessment group	*	*	*	*	*	*		*	*	*
Same method of ascertainment for cases and controls	*	*	*	*	*	*		*	*	*
Non-response rate										
Same rate for both groups										
Non-respondents described										
Different rates and no designation										
Total quality score	4 (M)	4 (M)	4 (M)	4 (M)	4 (M)	6 (G)		4 (M)	4 (M)	4 (M)

## Results

### Study selection

The outcomes of the search strategy are outlined in the PRISMA flow chart (Figure 1) (7). The search of the six databases revealed 2,713 studies. Following the removal of 538 duplicates, 2,175 were retrieved. Of these, 2,147 irrelevant studies were eliminated based on the title and the remaining 28 articles were retrieved for abstract reading. The abstracts of 28 articles underwent review, leading to the exclusion of 22 articles. Thus, six studies were chosen. Subsequently, three articles met the inclusion criteria after the full-text evaluation. Consequently, a total of 10 studies, all retrospective in nature, were incorporated into this systematic review.

### Study characteristics

The included 10 studies were individually examined and data regarding the author's name, study design, sample size, the participants’ type of occlusion, and methods of assessment were stored in a Microsoft Office Excel spreadsheet (Table 1). These data were then shared with the senior reviewer to streamline and finalize.

Of the included studies, eight studies defined the participant's occlusion. Henry (8) included participants with normal occlusion, Class I with premolar extraction, Class II division 1, Class II division 1 subdivision, and Class II division 2. Lamons and Holmes (9) divided the participants into three groups where group 1 had normal occlusion, group 2 had post-normal occlusion, and group 3 had prematurely extracted deciduous molars. Two studies (10, 11) did not define the type of occlusion. Dahlquist et al. (12) categorized the study participants into two groups; group 1 had normal occlusion and group 2 was the treatment group. Hansen et al. (13) included normal occlusion participants who were untreated. Junqueira et al. (14) included both normal occlusion and Class II division 1 malocclusion and they were categorized into groups 1 and 2, respectively. Vermeulen et al. (5) included Class I and Class II division 1 malocclusion (non-extraction) and were categorized according to the time point with T1 denoting pretreatment, T2 denoting post-treatment, and T3 denoting after the removal of a retention appliance. Scanavini et al. (15) included participants with Class II division 1. A study by de Oliveira Viganó et al. (16) included participants with Class I, II, and III malocclusions which were further divided into five groups depending on whether they were skeletal or dental.

Eight out of the 10 studies (8, 9, 11–16) assessed the rotation of various teeth and gave a quantitative outcome and the other two studies (5, 10) had a qualitative outcome. Vermeulen et al. (5) measured the rotation quantitatively but the final results were not quantitative as they evaluated the reliability of the method of assessment of rotation of all permanent dentition.

Of the included studies, eight studies used dental casts to measure the degree of rotation (8–11, 13, 15, 16, 18) while two studies used a photocopy of dental casts to measure the degree of rotation (5, 14). Eight studies (8–10, 12, 14–16) assessed the rotation of molars, but Hansen et al. (13) assessed the rotation of permanent maxillary mandibular first molars and maxillary first premolars. McMullan and Richardson (11) assessed the rotation of premolars and Vermeulen et al. (5) assessed the reliability of the measurement of rotation of all permanent teeth.

Eight studies (8–14, 16) used a line passing through the mid-palatal raphe as the reference line and two studies used a line on the mesiobuccal cusp and distobuccal cusp as the reference lines (5, 15) (Table 1). No gender differences were found.

### Risk of bias of individual studies

All 10 studies included both sexes. The Newcastle–Ottawa scale was used to assess the risk of bias (Table 2) (19). The risk of bias for nine studies was rated as “moderate” (5, 8–11, 13–16) while Dahlquist et al. (12) was rated as “good.”

### Results of individual studies

The included 10 studies were individually examined, and the data regarding the author's name, study design, sample size, the participants’ type of occlusion, methods of assessment, and outcome were stored in a Microsoft Office Excel spreadsheet (Table 1). These data were then shared with the senior reviewer to streamline and finalize.

The skeletal Class II and Class III groups showed a similar mean position for the first molar. Upper molar rotation mainly occurred in the dental Class II patients, with a higher mesial rotation angle of 78.6°.

Henry (8) showed that there is a tendency for mesial movement of the upper first permanent molar in Class I and Class II malocclusions and the outcome was −10.3° (R), 12° (L) for the Normal group; −19.5° (R), 15.5° (L) for the Class I extraction group; 20° (R), 17.6° (L) for the Class II division 1 group; 18.8° (R), 16° (L) for the Class II division 1 subdivision group; and 19.5° (R), 16.9° (L) for the Class II division 2 group. Lamons and Holmes (9) measured the rotation of the permanent upper molars using Friel's method and concluded that the mean rotation of the molars was 61° ± 4°.

Dahlquist et al. (12) concluded that by utilizing the reference lines from the Friel, Henry, and Orton methods, the mean angle for the rotation of the upper first molar was 4.3° for Angle 1, 2.5° for Angle 2, and 2.0° for Angle 3, respectively. Hansen et al. (13) found the average mesiolingual rotation of the maxillary molars, measured through the buccal cusp tips, was 14.08° for the right molars and 12.76° for the left molars relative to the midline. For the mandibular molars, the average mesiolingual rotation was 19.83° for the right molars and 21.88° for the left molars relative to the midline.

In the study by Junqueira et al. (14), it was concluded that individuals with Class II division 1 malocclusion exhibited greater mesiopalatal rotation of the maxillary first molars. The mean rotation angles in Group 1 were Angle 1: 10.07°, Angle 2: 61.54°, and Angle 3: 12.69°, and Angle 1: 14.97°, Angle 2: 57.44°, and Angle 3: 5.93° in Group 2.

de Oliveira Viganó et al. reported that the dental Class II group had the highest mean mesiopalatal rotation of the upper first molar (78.6°) with a greater frequency, followed by the skeletal Class II group (74.95°), skeletal Class III group (73.83°), and Class I group (71.9°). The average rotation of the anterior teeth ranged from 3.3° to 11.0° (5, 20).

Vermeulen et al. (5) stated that the incisors exhibited the highest intraclass correlation coefficients (ICCs), ranging from 0.876 to 0.991. The molars had the lowest ICCs, ranging widely from 0.430 to 0.935.

## Discussion

This systematic review aimed to collate the various assessment methods for the rotation of teeth to assess their consistency and clinical implications. Since the included studies were observational and cross-sectional, the NOS was selected to assess the risk of bias. The NOS provides structured evaluation across key domains, such as the selection of study groups, comparability between groups, and the accuracy of outcome assessments, since tooth rotation classification is inherently complex, with studies using varied methods, populations, and outcomes. Nine of the 10 studies (5, 8–11, 13–16) were rated as “moderate” quality, indicating reasonable methodological soundness but with limitations such as inconsistency in assessment methods and variability in outcome measurements. In contrast, the study by Dahlquist et al. (12) was rated as “good” quality, reflecting its adherence to higher methodological standards, including participant selection and clear control of confounding variables.

The rotation of anterior teeth, particularly the maxillary incisors, is typically more consistently measured and plays a critical role in both esthetics and function (5). Maxillary incisors exhibit higher degrees of rotation, ranging from 7.4° to 20.2° (5). While untreated rotations can lead to compromised smile esthetics, whether a greater quantum of rotation would lead to a greater impairment of esthetics would be of clinical and research interest. The literature is restricted to descriptions of the maxillary arch for the anterior teeth. Premolar rotations in the maxilla and mandible, while generally more reliable to measure than molar rotations (with ICC values of 0.876–0.991), still encounter challenges due to inconsistencies in reference lines and their correlation with arch shape (5, 21). Despite being less emphasized in clinical practice, the correction of premolar rotations is vital for preventing arch crowding and occlusal interference (3, 5, 20, 22, 23).

The measurement of molar rotation has exhibited considerable variability, primarily due to differences in reference points and measurement techniques. The studies by Dahlquist et al. and Henry employed varying methods to assess molar rotation, thus making direct comparisons among the findings clinically difficult (8, 12, 13). Furthermore, reproducibility remains inconsistent, particularly in molar assessments, as evidenced by low ICC values ranging from 0.430 to 0.935. These discrepancies highlight the urgent need for a standardized method in both research and clinical settings to accurately measure molar rotations. Reliability in these assessments is critical; the low ICC values indicate a lack of consistency over time and among different observers. This lack of reliability compromises the clinical applicability of findings, making it difficult to form cohesive treatment plans (5, 9, 11). Correcting molar rotations is crucial for maintaining occlusal relationships and avoiding complications such as occlusal interferences and uneven force distribution (13). If not stabilized adequately during the retention phase, molars are prone to revert to their original positions. For example, the rotation of the mesiopalatal cusp in molars is particularly critical in Class II malocclusions, where uncorrected rotations can significantly hinder long-term treatment success and stability (16, 21, 24).

Among the malocclusions, the skeletal Class II and Class III groups showed a similar mean position for the first molar. In the dental Class II patients, there was pronounced upper molar rotation, with a higher mesial rotation angle of 78.6°. This aligns with the findings of Henry (8), who noted a tendency toward mesial movement of the upper first permanent molar in Class I and Class II malocclusions. The specific outcomes in Henry’s study included values of −10.3° (right) and 12° (left) in normal cases, with more significant rotations observed in the Class I extraction cases (−19.5° right, −15.5° left) and various Class II malocclusions [e.g., Class II division 1: 20° (right), 17.6° (left); Class II division 2: 19.5° (right), 16.9° (left)]. Lamons and Holmes (9), using Friel's method, measured the rotation of the upper permanent molars and found an average rotation of 61° ± 4°. These findings suggest that upper molar rotation varies across different malocclusion types, with significant mesial movement in Class II cases (12–14).

A meta-analysis could not be performed in this review because there was no more than one study comparing tooth rotations in malocclusions. Further, the included studies employed diverse techniques, reference lines, and assessment tools, making it impossible to directly compare mean rotation values.

Orthodontically rotated teeth are prone to relapse after treatment, which is a common and unpredictable challenge in orthodontics (5, 25). Oppenheim highlighted retention as one of the most difficult problems in the field, while Reitan (26) observed that rotated teeth often tend to return to their original positions once appliances are removed. Quantifying rotational relapse remains ambiguous, though Andrews (27) emphasized that the absence of dental rotation is critical for achieving normal occlusion (28). Rotations are commonly associated with both crowding and excess space in the dental arch, and the precise measurement of rotation is crucial for further research (28, 29). Correcting rotational discrepancies, especially in posterior teeth such as molars and premolars, is vital for proper occlusion and alignment. Posterior tooth rotation, particularly in the maxillary first molar, affects space distribution and can complicate anterior alignment (16). Studies by Liu, Melsen, and Henry indicate a tendency for molars to rotate mesially, often exacerbated by habits such as thumb-sucking and abnormal swallowing patterns, which can lead to dental crowding and impaired occlusal stability (5).

The importance of tooth rotation has clinical implications in all fields of dentistry, including orthodontics. Rotated teeth can complicate prosthetic restorations by disrupting occlusal alignment, requiring adjustments in crown preparation to ensure proper axial alignment and retention. In implant dentistry, orthodontic pre-alignment may be necessary to create space for optimal implant placement, particularly for rotated molars (30). Rotational misalignment also poses challenges in endodontic treatments, altering the orientation of root canals and increasing the risk of perforations, missed canals, or inadequate apical sealing (31, 32). In addition, rotated teeth can exacerbate periodontal issues by promoting plaque accumulation due to irregular surfaces, leading to traumatic occlusion, gingival recession, and attachment loss (33). In restorative treatments, achieving optimal esthetics with veneers or composites on rotated anterior teeth requires careful planning to ensure natural alignment and appearance (34, 35). Incorporating these multidisciplinary perspectives into the classification of tooth rotation enhances clinical understanding across specialties, promoting more comprehensive and effective treatment strategies for both functional and esthetic outcomes (33, 35).

However, a persistent challenge across the assessment of molar, premolar, and anterior tooth rotations is the variability in measurement techniques. Different studies frequently employ various reference lines, leading to inconsistent results. Although advancements in digital tools and photographic analysis have enhanced reproducibility, the lack of standardization remains a significant issue, especially for posterior teeth (5, 21). Consistency is also a concern, as differing reference points and techniques lead to varied results, complicating our understanding of molar rotation's clinical implications. Validity, which assesses whether measurements accurately reflect the intended discrepancies, is also at risk without standardized protocols, potentially leading to misinterpretation of the significance of molar rotations in treatment outcomes (10, 13). While maxillary teeth rotations have been studied extensively, mandibular anterior tooth rotations have received less attention, leading to a gap in understanding their implications for occlusion and esthetics (24, 25, 34, 36).

The limitation of this systematic review was that only English language studies were included. Further, the hand search was restricted to four orthodontic journals. Non-orthodontic journals were not hand-searched. A meta-analysis could not be performed as there was not more than one study with quantitative data that could be collated. Future research should prioritize the development of standardized measurement protocols and explore the long-term effects of rotational corrections on treatment outcomes. Studies that focus on the relationship between the quantum of rotation and relapse, the effect of various interventional procedures such as a circumferential supracrestal fiberotomy, and the influence of the retention protocol on relapse are required. By emphasizing customized retention strategies during the post-treatment phase, orthodontic practitioners can enhance their ability to prevent relapse and achieve more predictable, stable, and esthetically pleasing results. Thus, the advancement of standardized methodologies is essential for improving the clarity and applicability of research findings in orthodontics, ultimately benefiting clinical practice and treatment outcomes.

## Conclusions

1. Variability in measurement techniques and a lack of standardized protocols to assess the severity hinder reliability when assessing tooth rotation and complicate treatment planning.
2. There is no adequate classification system to assess the rotation of mandibular dentition and to assess the rotation of anterior teeth.
3. Existing systems for posterior teeth need to be standardized and have clinical utility to be widely accepted.
4. Focusing on consistent measurement methodologies and retention will improve orthodontic predictability and stability, enhancing patient outcomes across orthodontics, prosthodontics, endodontics, and periodontics.

## Data Availability

The raw data supporting the conclusions of this article will be made available by the authors, without undue reservation.
